# From schools of thought to an ecology of practices: Categorizing circular economy's futures

**DOI:** 10.1111/jiec.13564

**Published:** 2024-10-08

**Authors:** Ryan Nolan, Esmaeil Khedmati Morasae, Mike Michael

**Affiliations:** 1 Department of Management, University of Exeter Business School, Exeter, UK; 2 School of Management, University of Bradford, Bradford, UK; 3 Department of Sociology, University of Exeter, Exeter, UK

**Keywords:** circular economy, ecology of practices, futures, practice theory, sociology of expectations, sustainability transitions

## Abstract

In response to pressing societal challenges, scholars are increasingly focusing on research aimed at fostering sustainable futures. We contribute to that discussion by theorizing the circular economy (CE) as an “ecology of practices.” The ecology of practices concept helps to make sense of a developing field that has been heavily practitioner-driven. Through an analysis of the diverse CE practices in analytical and operational contexts, we investigate the roles, disciplinary influences, and visions for the future and categorize their trajectories. Drawing on the sociology of expectations, we consider the articulations of CE in practice, advocating for inclusive dialogue among stakeholders and collective engagement with ontological multiplicity in shaping CE futures. We propose a framework that contributes to broader debates in organization and management studies, emphasizing the significance of everyday practices in shaping sustainable futures beyond the realm of CE. In so doing, we focus on unpicking how sustainable futures are variously enacted as a way of enabling collaboration that might otherwise be hindered by disciplinary obligations.

## INTRODUCTION

Scholars have grappled with addressing today's grand challenges in pursuit of more sustainable futures, particularly over the past decade (Ferraro et al., 2015; Howard-Grenville & Spengler, 2022). Conceptual and empirical research has considered how the field can contribute to climate change (George et al., 2016), planetary boundaries (Whiteman et al., 2013), and the UN Sustainable Development Goals (Williams et al., 2019) through business strategy and theory building. Some of the most pressing issues of our time stem from the climate and ecological crises. At the same time, scholars have emphasized the role of practice as the means through which grand challenges will ultimately be addressed (Ackermann et al., 2024). That is to say, while knowledge of and about these challenges helps us understand the situation, better futures and social realities will be brought about through the coordination and execution of plans and solutions of a variety of “future-making” practices (Wenzel et al., 2020; see also Schatzki, 2010).

In this respect, this paper aims to explore the circular economy (CE), drawing on the philosopher of science Isabelle Stengers (2005) to adopt an “ecology of practices” view of the interaction of different perspectives and practitioners. This approach not only helps to bridge the gap between theory and practice but also underscores the operationalization of CE principles, demonstrating a pathway for effectively engaging interdisciplinary insights in real-world settings. As an emerging field that has been driven by its strong practice dimension across policy and industry, alongside ambitions to foster sustainable production and consumption often associated with SDG 12, CE functions as a particularly apt site for our analysis.

Our study is not interested in simply laying bare the (possible intractability of the) diversity of CE enactments. Rather, we aim to trace the diverse but specific articulations of temporality within CE (ranging from schools of thought to local practices) to suggest how these might be organized, or “staged,” in generative juxtaposition and useful dialogue that might otherwise be hindered by disciplinary norms and obligations. In presenting this aspiration, we also need to be aware of, and circumspect about, the fact that we too are enacting a future temporality: This is important as to engage in an “ecology of practices”—a bringing together of those practitioners of CE—is also to ensure that one does not impose one's own particular model of organization or staging along with its associated promises.

In adopting and advocating for an ecology of practices perspective, we also contribute to an emerging debate in organization and management theory around the role of theory-building as a normative (Hanisch, 2024; Wickert, 2024) future-making practice (Gümüsay & Reinecke, 2021; Wenzel et al., 2020) in itself, but rather than concurring with the operationalization of the performative effects of theory, we come down in favor of a subtler approach (Horner et al., 2024) rooted in the coming together of everyday practices (Ackermann et al., 2024).

The remainder of this paper is structured as follows: We first introduce the CE and describe our methodology. We then present an overview of various analytic approaches to CE as represented in the extant literature, drawing out their core epistemic characteristics, which includes an account of practitioner operationalizations of CE to emphasize the key practical conditions that are faced. We then address how the different versions of the world—ontologies—can be brought together, considering the conditions by which a fruitful collective engagement with ontological multiplicity can be achieved in the case of CE futures. Subsequently, we unpack a number of these futures of CE before discussing how an ecology of practices approach can productively bring them together. In the conclusion, we reflect on the implications of our study for both CE research and practice, as well as broader considerations for addressing grand challenges through an ecology of practices lens.

## CONTEXTUAL FRAMING: THE CIRCULAR ECONOMY

Many of today's challenges such as environmental change, overproduction, and social inequality are intricately linked to our relationships with the material world. The way we produce and consume things—systems vital to sustaining life and those that satisfy more temporary desires—plays a particularly important role in defining the nature of this relationship. In many respects, the past two centuries can be characterized by humanity's efforts to refine a specific approach to production and consumption, where, by and large, finite materials are extracted from nature, manufactured into products, shipped to consumers, used for a time, and discarded as wastes (Stahel, 2016). As materials flow in one direction from point A to point B with little return, this model of production and consumption has been conceived as the “linear economy.”

The linear economy is an unsustainable system that disregards the value embedded within products already in circulation and precludes any potential value of those products by removing them from the system as wastes that are sent to landfills or incinerated. In so doing, it has led to increasing quantities of greenhouse gases released into the atmosphere and driven inequality across the globe (Neves & Marques, 2022). Recent years have seen various frameworks proposed that attempt to reimagine how we organize our relationship with things in ways that are more sustainable and equitable. In particular, the linear economy has been contrasted with CE, which has emerged over the past few decades and is informed by several “schools of thought” that we discuss below. While definitions abound (see, e.g., Kirchherr, et al., 2023; Yang et al., 2023), at its core the CE centers around designing out waste and pollution from industrial systems; maintaining products and materials in circulation at their highest values for longer through mechanisms such as reuse, repair, and remanufacture; and regenerating natural resources (Ellen MacArthur Foundation, 2023; Stahel, 2016).

A core strength of the CE concept has been its reception by practitioners (Corvellec et al., 2021, p. 422), interested in implementing its principles in industrial contexts as a mechanism to achieve greater sustainability. However, CE is increasingly recognized as a burgeoning scholarly field in its own right (Kirchherr et al., 2023; Urbinati et al., 2023) and is receiving increased attention from academics and researchers. That being said, a great deal of the literature focuses on technical dimensions of circularity, such as integrating reverse logistics like recycling into value chains or producing novel business models to facilitate and drive innovative circular solutions. While these lines of inquiry are certainly vital to the technical and operational dimensions of scaling and implementing CE, an emerging body of scholarship highlights the need for deeper analysis of its social (Corvellec et al., 2021; Monciardini et al., 2024), theoretical (Korhonen, J., Nuur et al., 2018), and political and organizational (Corvellec, 2019) dimensions, dimensions which ultimately affect the interpretation of CE at local levels.

The lack of discussion of the social in CE discourse is not a weakness per se, but an externality of an emerging field that is replete with many knowledge gaps yet to be filled. Nevertheless, our intention is to engage with the ways in which sometimes competing views of CE might fruitfully intersect. In particular, we are interested in an inclusive approach that embraces not only conceptual and technical accounts of CE but also local operational practices (the things that are done to bring about circular futures). By thinking about how these diverse voices can be “brought together,” we hope to open up productive possibilities of the actualization of CE, whether this be cogent, piecemeal, or provocatively fragmented. Here, attention to the social is pivotal.

## METHODOLOGY

Our study adopts an “ecology of practices” approach, drawing inspiration from the work of philosopher Isabelle Stengers (2005), to examine the CE through a social lens. This approach allows us to explore the diverse perspectives and practices within the CE field, combining theoretical analysis with empirical investigation through a qualitative, interpretive lens.

Our data were gathered from two primary sources: academic and practitioner literature on CE and case studies of CE implementations published online. Rather than conducting a systematic review, we adopted a more ad hoc, narrative approach. This strategy allowed us to focus on highly cited papers from the last decade, emphasizing influential research that addressed key topics such as resource efficiency, waste reduction, recycling technologies, sustainable business models, and CE policy frameworks. The aim of this approach was not to exhaustively cover and map the extant literature but to identify schools of thought relevant to constructing a framework based on insights from the sociology of expectations. This flexible approach facilitated the identification of major trends while also capturing the interdisciplinary nature of CE and offering insights into anticipated timelines for transitions across different sectors.

To analyze our data, we devised a framework inspired by the sociology of expectations (Borup et al., 2006; Brown & Michael, 2003). This framework guided our examination of CE futures across several key categories, including the formulation of the future, site of transition, distance of future, speed of approach, impediments to progress, and key actors. It enabled us to systematically compare and contrast different visions of CE futures across various schools of thought and local practices.

Our analytical process unfolded in several stages. We began by characterizing various analytical approaches to CE and then analyzed how CE is operationalized in practice. We examined how CE futures are enacted by different advocates and practitioners, and finally synthesized our findings to develop an ecology of practices view of CE.

The validation of our framework followed an interpretive process, reflecting the qualitative nature of our study. Authors 1 and 2 iteratively devised the categorization of CE practices and their possible trajectories, informed by their analysis of the literature. This categorization was then presented to author 3 for further discussion, where multiple interpretations were explored, debated, and refined. This internal, dialogical process of validation aligns with the epistemological stance of the study, emphasizing interpretation and meaning-making rather than objective categorization. As such, the framework is not intended as a definitive taxonomy of CE futures but rather as a heuristic tool for exploring the diversity of perspectives and practices within the field.

## ON THE MULTIPLE VERSIONS OF CIRCULAR ECONOMY

Some scholars now argue that CE has evolved into an academic field in its own right (Kirchherr et al., 2023; Urbinati et al., 2023). This transition is evident through the emergence of dedicated epistemic communities or schools of thought within CE, each unified by increasingly cohesive methodological practices and norms (Kirchherr et al., 2023; Urbinati et al., 2023). The conceptual forerunners of CE are well documented and attempts have been made to draw them together as a way of unifying the academic discourse (Borrello et al., 2020). Despite this, there are still doubts regarding the boundaries of CE and what it means in theory and practice. Namely, due to its mainly practitioner-driven history of development, some see it as superficial, as patchy, and even as an empty signifier, that has, perhaps paradoxically, not been well-translated toward practical implementation (Korhonen, J., Nuur et al., 2018; Korhonen, J., Honkasalo et al., 2018).

Notwithstanding this, there are a number of distinct epistemic communities of CE that are pushing the concept forward in several directions (Borrello et al., 2020; Kirchherr et al., 2017). What we present here is not an exhaustive list but a sample of 10 practices as present in extant literature. The main aim of this section is to sketch out the key elements that typify an array of versions—epistemic communities—of CE. This sets the scene for later exploring the sorts of future that each version of CE enacts.

The first epistemic community centers on a belief that CE should entail a systemic transition (De Angelis, 2022; Desing et al., 2020; Bicket, 2020; Blomsma et al., 2023). For instance, using a multi-level selection theory, Figge et al. (2021) argue that a system or group of firms, and not individual firms, should be the focus of CE interventions. The reasoning here is that a cohesive group of firms has a higher survival chance in a transition toward CE (this is grounded on individual firms not competing). Desing et al. (2020), as another example, argue that CE at micro levels should not be blind to systemic resource boundaries of the planet; otherwise, it will lead to more environmental destruction. Similarly, Iacovidou et al. (2021) argue that the CE community should address five dynamic interconnected sub-systems that need to be considered for supporting complex transitions to CE; namely, resource flows and provisioning service; governance, regulatory framework, and political landscape; business activities and the marker; infrastructure and innovation; and user practices. Such a complex systems perspective indicates that a CE transition involves many diverse groups of materials, actors, industries, governance structures, and environmental issues, interacting at different levels and working differently. Therefore, managing or steering change toward greater circularity requires a systems approach—one that is sensitive to this complexity.

The second community revolves around the idea of decoupling as it relates to the relationship between environment and economy and focuses on the fact that CE might help (or fail) to reconcile economic growth and environmental sustainability (Kirchherr, 2022). Many CE scholars agree that such a decoupling is possible, but a growing camp criticizes such a stance and holds that without a social and critical approach, there is a high possibility that CE will lead to unsustainability or, in the best scenario, partial sustainability. There is a growing discourse that argues CE is based on a depoliticized techno-modernist ideology that is blind to structural challenges to sustainability transitions (Corvellec et al., 2021; Hobson & Lynch, 2016; Merli et al., 2018) and this matter will render CE a failed concept. A spin-off from such discussions within this school is the emergence of a chasm between those who favor a growth-based CE and those who support a post-growth CE (Bauwens, 2021; Kirchherr, 2022).

The third CE community relates to transition studies. This emerged from sustainability studies before CE was a widespread concept but nowadays comprises a distinct epistemic community. CE transition studies share similarities with the systemic approach described above, but it is more sensitive to socio-technical aspects of transitions. There is a growing literature that argues that multi-level perspective (MLP) theory, which is an established theory in transition studies, can help transition to CE at micro, meso, and micro levels (Ahmadov et al., 2023; Ahmed et al., 2022; Miemczyk et al., 2022; Geels, 2011; Trevisan et al., 2023). Overall, MLP theory provides a structured framework for studying and facilitating sustainability transitions by recognizing the importance of both incremental niche innovations and broader landscape changes in challenging and transforming established socio-technical regimes.

The fourth community relates to concerns regarding the link between CE and sustainable development. Interestingly, one of the most cited articles published in the CE community belongs to Geissdoerfer et al. (2017) who proposed CE as a paradigm for sustainable development. This school has carved out a niche and papers are being published increasingly promoting a “sustainable” CE (Jaeger-Erben et al., 2021; Velenturf & Purnell, 2021).

The fifth epistemic community works on circular business models (CBMs) and related value chains (Urbinati et al., 2017, 2021; Unal et al., 2019; Bocken et al., 2016). CBMs are one of the most vibrant topics discussed in CE, not least because some scholars (e.g., Kirchherr et al., 2017) have fundamentally defined CE as based on CBMs. They argue that CBMs need to be taken seriously; otherwise, CE devolves into a steering wheel with no driver behind it. Here, a CBM can be defined as a logic through which a business or company creates and exchanges values with its customers and stakeholders while aiming to keep the relevant material/products in circulation for as long as possible at their highest value and quality (Urbinati et al., 2021).

The sixth community sits in conjunction with the former but focuses on the design of products that can go through the value and supply chain in CBMs. This school reflects a more engineering-focused mode of thinking and investigates the type of symbioses possible among industries and related products to produce a coherent circular supply chain. Scholars working in this area are particularly concerned with the design of processes, products, and industries that allows CBMs to generate value from life-prolonged products and services where resource loops are closed, product disassembly is guaranteed, and waste is obsolete (Bocken et al., 2016; Burke et al., 2021; De Angelis et al., 2018; Mestre & Cooper, 2017).

The seventh community relates to an increasing recognition of the importance of public policy as a driver of circularity. To be precise, there is a mounting consensus now that the CE transition requires direct government intervention in regulation, infrastructure, education, and other factors (Milios, 2018; Hartley et al., 2020; McDowall et al., 2017; Milios, 2021). Some policy-related interventions proposed for CE include more robust standards and norms in production, expansion of circular procurement, tax relief for circular products, liberalization of waste trading and its facilitation through virtual platforms, support for eco-industrial parks, and awareness campaigns (Hartley et al., 2020). There is also a growing interest in examining a comparative CE policy landscape across the globe, especially between Europe and China as pioneers (McDowall et al., 2017).

The eighth community is concerned with the organizational aspects of the CE transition. There is a growing literature regarding the strategies organizations are adopting (or failing to do so) to become circular. Organizational issues like resources, capabilities, dynamic capabilities, stakeholders, and circular leadership are attracting interest in the CE literature (Khan et al., 2020; Kohler et al., 2022; Seles et al., 2022). This community argues that the CE transition will not be smooth if organizational issues and enablers are not well understood (Seles et al., 2022).

The ninth concerns the measurement of circularity. This community focuses on the need for benchmarks by which to judge the degree of transition to circularity within a nation, a region, or a business. A large number of CE indicators are proposed for different scales and levels (micro, meso, macro) and dimensions (environmental, economic, social) of the CE transition (Calzolari et al., 2022; Cayzer et al., 2017; de Oliveira et al., 2021; De Pascale et al., 2021; Moraga et al., 2019). There is a growing literature here arguing for a need for integrative frameworks of taxonomies to help make the use of these indicators easier for policymakers and practitioners (Cagno et al., 2023; Saidani et al., 2019; Urbinati et al., 2017).

Finally, the 10th community in CE research relates to mathematical/statistical modeling and simulation of CE intervention and systems. As CE is seen as a systemic phenomenon or an intervention in a complex system, there is a growing interest in simulating CE interventions and policies to gain a better understanding of the intended and unintended consequences such intervention might bring about. Complex systems science methods like system dynamics, network analysis, and agent-based modeling are thus gaining traction, both as ways to generate new insight but also as ways to make sense of CE practices at scale (Franco, 2019; Guevara-Rivera et al., 2021; Guzzo et al., 2022; Koide et al., 2023; Lange et al., 2021; Walzberg et al., 2023).

In this section, we have set out a number of versions—or epistemic communities—of CE, focusing on their basic characteristics. There are two points to note here. First, tacit in these versions are enactments of the future—how CE will evolve as a set of practices, policies, interventions, theories, and so on. There are expectations enacted that point to how CE might become established and integrated into broader social and economic structures. There are implicit promises about the advantages that CE, actualized in various ways, will afford. We will return to detail these below. Second, so far we have dealt only with what amounts to analytic accounts of CE. To be sure some of these address matters of local practice (and of course involve their own working practices), but if we are to pursue an ecology of practices in relation to CE, we need to make space for less professionally “analytic” voices, in particular those of practitioners. In the next section, therefore, we present a few examples of the ways in which practitioners—those who are involved in implementing CE on the ground as it were—enact CE (and its futures).

## CE IN PRACTICE

The practitioner literature offers a lens through which to explore the challenges and potential of transitioning toward a CE experienced by organizations “on the ground.” This is useful in that it helps us better understand the general complications related to CE as a future-making practice. A recent *Harvard Business Review* article tells the story of Interface—a US-based organization that manufactures carpets (Atasu et al., 2021). Driven by a leadership team invested in transforming the company into the “first sustainable corporation in the world,” Interface spent a decade pursuing a lease-based business model to the detriment of company performance. Rather than abandoning the project, the organization took the time to understand the factors impeding their approach and came to a new sales-based model for modular carpet tiles. This is an indicative example of navigating the complexities of transitioning to CBMs, reflecting the dynamic interplay between actions taken in the present and future aspirations within CE practices over time.

Furthermore, the emphasis on CBMs within practitioner discourse underscores the highly iterative nature of CE practices, where ongoing experimentation and adaptation are essential for navigating the uncertainties of future transitions (Frishammar & Parida, 2021). By integrating these practitioner insights into the broader narrative of the CE and its futures, we gain a deeper understanding of how diverse perspectives and practices intersect to shape trajectories of sustainability and resilience. That is to say, the challenges encountered by companies like Interface underscore the importance of considering the future implications of current decisions (temporal trade-offs, see Hahn et al., 2010), aligning with the notion of embracing the “ecology of practices” perspective advocated in this paper. This perspective emphasizes the need for practitioners to recognize their responsibilities and the disciplinary commitments informing their accounts of CE, while simultaneously envisioning and shaping future trajectories (Stengers, 2005).

Despite the widespread recognition of the CE concept and its integration into various policies and business strategies, the reality paints a different picture. As reported in the *Circularity Gap Report 2024*—which has become a major source of reference among practitioner communities—global circularity has been on a decline, dropping from 9.1% to 7.2% over the past 6 years. This trend underscores the existence of two significant circularity gaps that are particularly interesting from the perspective we take here: one between the current level of circularity and the desired state, and another between the awareness of CE and its practical application (Fraser et al., 2024). As we confront the challenges of environmental degradation and resource depletion, it becomes increasingly evident that merely espousing the principles of circularity is not enough; concerted action is needed to bridge these gaps and pave the way for truly sustainable futures, where understanding how such futures are constructed becomes centrally important and as yet under-researched.

## ECOLOGY OF PRACTICES: CIRCULAR ECONOMY FUTURES

In considering the various approaches to, and commentaries upon, CE, there are obvious differentiations to be drawn. These range across, for instance: the political–economic “extent” of CE (whether it entails comparatively minor tweaks to governmental policy at one end vs. radical reconfigurations of social structures on the other) (Genovese & Pansera, 2021); relatedly, the extent to which the “economic” is privileged over the political or the environmental (Morseletto, 2020); the degree to which CE can be considered a “discipline” or a cogent research field (Kirchherr et al., 2023; Yang et al., 2023). However, there are also other dimensions to the various positions within CE that have received less explicit attention. These concern temporality as it applies both to the expectations and futures attached to the establishment of locally defined CEs and the expectations and futures associated with the developing research field (as a scholarly, policy-oriented initiative).

Beyond these differentiations, the temporal aspects of CE are intricately linked, particularly as the field continues to evolve. The emergence of CE as a practical economic-environmental assemblage (Korhonen, J., Nuur et al., 2018) is substantially mediated by ongoing research and commentary, including numerous diagnoses, overviews, and advocacies for particular approaches. Rather than viewing the areas of practice (analytic and practitioner) as separate, we propose a more inclusive process. This approach aims to incorporate—at least at the level of discourse—the various time frames faced by practitioners implementing CE as local activities and analysts characterizing CE as a “movement,” field, or phenomenon.

While being cautious of contributing to the proliferation of meta-commentaries, we aim to explore how this varied field might be conceptualized further in terms of various potential futures shaped by different analytic and practitioner actors. In this regard, we draw on Stengers’ notion of an “ecology of practices” (2005), where participants from diverse disciplinary backgrounds contribute their understandings of what the CE involves. These perspectives are informed by each participant's unique practices—whether in research or application—reflecting their distinct ontologies (Mol, 2002) of CE. This approach involves viewing CE both as a concept to be analyzed and as a practice to be implemented, enriched by the specific expertise and experiences of each contributor. As a model for collaboration, an ecology of practices thus aims to bring together analysts and practitioners who might be more or less engaged in the analytics aimed at, and practicalities and policymaking associated with, CE. Central here is that any interested actor can have a voice, which Stengers’ insists is grounded in a form of expertise that encompasses all relevant experience.

Stengers suggests that our perspectives are “partially shaped” by what she terms “obligations,” or “attachments,” which she describes as the forces that shape our thoughts and abilities (Stengers, 2005, p. 191). These attachments could be to the tools and methods we use, the technologies we apply, or the communities we are part of. However, these attachments are not fixed from the start; they develop as we interact and respond to others. In essence, Stengers argues that our roles and identities within our disciplines or professions—what she refers to as our “belongingness”—should not be seen as predetermined. Instead, they evolve over time, reflecting her belief, rooted in Whitehead's processual philosophy that all participants in a field are continually evolving, always in the process of change.

In tandem with attachments are what Stengers calls “responsibility,” that is, a pragmatic “paying attention” as best one can about a situation, where one is not beholden to some general principle. In the context of a debate or negotiation among practitioners, this means opening up one's imagination to others’ accounts and unfamiliar perspectives. In this admittedly idealized scenario, an ecology of practices promotes a commitment on the part of analysts and practitioners to other analysts and practitioners, to continued interaction in which all are given their due.

Critically, the process entailed in such intersections and the “staging” inter-relations itself remains emergent, that is to say, *up for revision*. To impose particular techniques of collaboration that are derived from particular traditions (e.g., political theory) is itself to endanger the process of mutual engagement.

As noted, for present purposes, however, we focus on a particular dimension of these specialist practices and analyses, and their inter-sections and inter-relations. Specifically, we are concerned with how they involve projections of the future, both the futures of CE as something being practiced in the world and the futures attached to the more or less cogent field of CE scholarship. As mentioned, we do not wish to differentiate these too rigidly, especially as we wish to have them inform one another. In this respect, we can speak, partly drawing on Stengers, of an “ecology of futures” in which different enactments (through practice) of the future are juxtaposed (Michael, 2017). These futures can be unpicked along a number of dimensions: distance of the future, the speed with which the future approaches or is approached, the agent that is moving or being moved toward the future, the valency of the future (hope/hype), and any impediments against its realization (Borup et al., 2006; Michael, 2000).

However, further, futures can be more or less “big” (in the sense of having a broad, perhaps even epochal, reach). On score, practitioners engaged in developing a version of CE within their particular enterprise or sector are engaged in a more or less “little future” that is manifested through practices as they unfold locally. By contrast, analysts who are attempting to characterize CE as a fundamental change in how economic and environmental considerations can be integrated enact a more or less “big future.” These too can be counterpointed and debated. Again paralleling Stengers’ account of staging the form of the ecology of practices, this focus on futures applies no less to the counterpointing of, and debate about, futures.

Taken together, the analysis of specific futures and the futures of their juxtaposition allows us to explore in hopefully sophisticated detail the future of possible resolution (a singular CE), balance (a stable pattern of CEs), or even fragmentation (competing versions of CE). By articulating CE in this way, we hope to contribute usefully to how the interdigitating practices and articulations of CE can fruitfully unfold.

## ON THE MANY FUTURES OF CE

We have attempted to outline a variety of practices^1^ related to the CE transition as extracted from the extant literature reviewed in Section 2, detailing and cataloging their characteristics across multiple dimensions. They are presented in Table Table 1 and range from systemic transitions and sustainable development to CBMs and mathematical/statistical modeling. Each epistemic community represents a form of practice (ways of doing things) that, we argue, takes up and addresses a specific aspect of CE development and therefore the trajectory of particular futures, and projects either minor or major transformations through their practice. Similarly, each practice implicitly or explicitly assumes certain time frames, which we have characterized as varying from short to long-term, with varying speeds of implementation (some futures will arrive faster than others) and distinct impediments that could slow down or block certain futures from emerging at all. Of course, this framework is intended as a heuristic device, used as a means to facilitate dialogue and reflection, and not a precise analytical model.

**TABLE 1:** Categorizing “future trajectories of practice” operating in the circular economy field.

Practice	Indicative literature	Formulation of future	Size of transition	Distance of future	Speed of approach	Impediments	Actors
Systemic transition	Figge et al. (2021)	Emphasizes a systemic transition, focusing on groups of firms or cohesive systems.	Major transformation	Medium- to long-term	Slow	Competing individual firms; lack of consideration for resource boundaries.	Groups of businesses, whole systems
Decoupling environment-economy	Kircherr (2021)	Aims to reconcile economic growth and environmental sustainability.	Major transformation	Medium- to long-term	Slow	Risk of unsustainability without a social and critical approach.	CE community, scholars, policymakers
Transition studies	Ahmed et al. (2022)	Utilizes multi-level perspective theory for socio-technical aspects of CE transitions.	Major transformation	Medium-term	Medium	Recognizes the importance of incremental niche innovations and broader landscape changes.	CE community, scholars, policymakers
Sustainable development	Geissdoerfer et al. (2017)	Positions CE as a paradigm for sustainable development.	Major transformation	Medium-term (various target states, 2030, 2050, etc.)	Slow (reversing or stalling in practice)	Diversity in perspectives on whether CE can serve as a paradigm for sustainable development or simply BAU.	CE community, scholars
Circular business models	Urbinati et al. (2017)	Focuses on circular business models and related value chains.	Minor to major	Short- to medium-term (new start-ups offering solutions vs. established organizations pivoting)	Fast (iterative)	Varied adoption and understanding of circular business models among practitioners and start-ups, difficulty to pivot for established organizations.	Businesses, CE community
Design for circular supply chain	Mestre & Cooper (2017)	Emphasizes engineering-focused thinking for circular supply chains.	Minor to major	Short- to medium-term	Slow (multinational, etc.) fast (local, community-based)	Challenges in designing processes, products, and industries for circular supply chains, including complexities in ensuring product disassembly, resource loop closure, and waste obsolescence by design.	Engineers, CE community
Public policy production	Milios (2018)	Advocates for direct government intervention in regulation, infrastructure, education, etc.	Major transformation	Medium-term	Slow	Requires concerted government intervention on various fronts that has not happened to date.	Government, policymakers
Organizational strategy	Seles et al. (2022)	Examines strategies adopted by organizations to become circular.	Minor to major	Short- to medium-term	Could be fast (depending on the desire of organizations, or slow if resistant)	Well-established organizational barriers in the literature, culture, infrastructure, desire from leadership, etc.	Organizations, CE community
Measurement of circularity	Moraga et al. (2019)	Focuses on developing indicators and frameworks to measure circularity.	Minor to major	Short-to medium-term	Fast	The diversity of proposed indicators and the absence of a consensus on a comprehensive framework may hinder effective measurement and benchmarking of circularity. Or, what futures are we trying to measure with metrics?	Policymakers, practitioners
Mathematical/statistical modeling	Guzzo et al. (2022)	Utilizes mathematical/statistical modeling for understanding CE complexity.	Minor to major	Short- to medium-term	Fast (modeled/artificial future)	Data availability and accessibility: models are only as good as their inputs.	Researchers, policymakers

For instance, “systemic transition,” the “decoupling” of environmental concerns from economic growth, and the discourse of “sustainable development” all herald (with the full meaning of that term) profound paradigm shifts, projecting futures that rely on substantial transformations. Anchored in theories of “socio-technical transitions,” these endeavors unfold over what we broadly characterize as medium to long-term horizons, navigating obstacles such as market competition and the precarious balance between profitability and ecological integrity.

The CBM, design for circular supply chain, and organizational strategy communities aim for minor to major changes, focusing on business models, engineering, and organizational strategies to become circular. These practices have short to medium-term horizons and vary in speed from fast to slow, depending on organizational willingness and the challenges of pivoting for established organizations. Here, the focal point is on enabling a future through a restructuring of the key actors and elements—both social and material—and their inter-relations. It implies a future mediated by direct intervention which, in turn, suggests a slower pace of transition given the socio-material impediments that will need to be overcome.

Public policy production, measurement of circularity, and mathematical/statistical modeling perspectives are critical for facilitating CE transitions, requiring major transformations. They focus on government intervention, developing circularity indicators, and understanding CE complexity through modeling. While these approaches aim for a medium-term future and can be fast in generating insights, they face impediments such as the need for comprehensive government action and the diversity of proposed indicators. By contrast to the previous array of viewpoints, here we have a future that is enabled by more knowledge—whether through policy development, measurement, or modeling. Accordingly, the future is indirectly facilitated through a reconfiguration of the epistemic context of CE, which implies an overall faster movement toward the future.

Actors involved in these practices span from groups of businesses and engineers to government and policymakers. Clearly, there is a temptation to unpick professional differences and commonalities, but this might be a tall order, given stakeholders’ respective practical and “disciplinary” obligations or attachments. What the emphasis on the temporal dimensions of their analytic practice does is open up the ways in which underpinning enactments of futures are divergently structured across these stakeholders. These are, arguably, less fraught than disciplinary attachments and might, it is hoped, facilitate collaborative efforts, or at least fruitful engagements, across various actors.

Embedded within these CE practices is a commitment to envisioning and bringing into reality more desirable futures (desirable is a relative term here, and can take on a host of characteristics depending on the practitioner. That is, it might equally refer to an environmentally just future or one premised on increasing return on investment). In reviewing a series of practitioner-authored case studies^2^ of research and development projects from small- and medium-sized enterprises, we find examples of orientation toward a diversity of different futures, crossing between temporally smaller and iterative, to larger more transformational change.

To provide a couple of illustrative examples, one project by the Alliance for Sustainable Building Products focused on developing CBMs (row 5) for the steel industry. The project emphasizes the importance of “tracking steel sections so that they can be easily reused in the future,” reflecting the practitioners’ strategic vision for, and relative importance of, the long-term sustainability of manufacturing processes, demonstrating their orientation toward implementation and tangible outcomes. The iterative nature of introducing niche innovations represents a slow transition; the distance of the future it envisions can be conceived in the short to medium term. That is, while a total transition from linear to circular will not be realized through individual acts (that is a major transformation), the evolution of individual business models represents steady progress from the business and CE communities.

On the other end of the spectrum, some practitioners aspire toward futures where economic prosperity aligns with environmental stewardship, “decoupled” as it were, from the environmental degradation of the linear economy. This represents a major transformation as we shift from the status quo to an entirely new system. Emphasis on aligning circular activities with the principles of sustainable development (row 4) reflects this aspiration, as organizations such as MineLOOP endeavor to “encourage the [broader] understanding of environmental impacts in the circular economy space.” Their efforts underscore the importance of reconciling competing priorities and fostering connections between economic and ecological imperatives in shaping the future of CE practices. Interesting in the context of sustainable development approaches is the symbolic marking of futures, written into legislation and international treaties such as the Sustainable Development Goals, with target dates of 2030, 2050, and so on. These slower but bigger futures, which are ever-shifting in practice, represent roadmaps to uncertain times. It is important to understand how faster but smaller future-making practices contribute to their arrival, and more so, how they might be connected.

By integrating these practitioner perspectives within a broader ecology of CE practices, including the analytical practice of academics, we gain deeper insights into the complexities of future projections that can emerge from CE as a developing field. These practitioner-oriented futures contribute to shaping the trajectory of CE initiatives and the broader but continuously evolving discourse surrounding sustainability transitions, but how might we do this, in practice, in a way that also minimizes the potential pitfalls of academic–practitioner collaboration?

## TOWARD AN ECOLOGY OF PRACTICES APPROACH

In the foregoing, we have pointed to a number of differences in the enactments of the future, notably in terms of the formulation of the future, the site of transition, the distance of the future, the speed of approach, impediments to progress, and key actors involved in the transition. We can note that these parameters, while pragmatically analytically useful, are also in actuality blurred. Indeed, we might say they are emergent as the various approaches we have noted interact and intersect with one another. However, what these differentiations do highlight is the way that different analysts are necessarily attached to, and entangled with, various obligations to professional standards whether these be, broadly speaking, theoretical or empirical. When juxtaposed with the practices of practitioners, we can point to a rather different differentiation. For practitioners, it is the immediate exigencies of accomplishing small-scale enactments of CE that are uppermost. For these actors, the future can be said to be “little” (Michael, 2017) in the sense of being delimited in time and space and attached to immediate and local practices (that are often embroiled with, and responding to, immediate and local challenges and opportunities that, say, the introduction of a new business model could feasibly address). This contrasts with the “big” futures projected by the various schools of thought discussed. These big futures describe a much more extensive temporal and spatial field in which CE manifests, at one extreme, as epochally pre-eminent.

However, let us pause here; the very practice and process of formulating a big future for (along with the challenges that must be overcome) CE is itself a “little future.” As Michael points out (in relation to the fields of public understanding of/public engagement with science and technology), the work of producing these big future accounts is part of the “business as usual” of academic work. In this respect, analysts are entangled in disciplinary debates and technical analyses that at once reflect their professional obligations and their local, everyday practices. Bringing practitioners into the picture, with their ongoing attempts to operationalize CE in the context of numerous contingencies, foregrounds how analysts likewise face contingencies, that is, emerge out of the nexus of conditions in which they are embedded. Brought into closer proximity with practitioners, and also other analysts, within an ecology of practices and futures, there is a prospect that all recognize the conditional and mutable status of their own practices and procedures in those of others.

Put otherwise, this mutual recognition opens up the prospect of enabling responsibility in the sense outlined above; that is to say a pragmatic “paying attention” as best one can about a situation, where one is not beholden to some general principle or longstanding obligation. In the context of a negotiation among analysts and practitioners, this means opening up one's imagination to others’ accounts and unfamiliar perspectives. In addition to our suggestion that there is a formal commonality in, and mutual recognition of, the contingency of enactments of “big futures” in the “little futures” of local everyday practices, we can also point to more substantive connections. There are shared among the practitioners and analytic communities generic structures of future enactments. At the most basic, there is a commitment to a “better” or more “desirable future” (Gümüsay & Reinecke, 2021), a frustrated sense of urgency, and an alertness to impediments.

## CONCLUSION

We are acutely aware that the discussion immediately above is characterized at once by high optimism and low specificity. This is intentional for five reasons. What the ecology of practices offers is a focus on the possible which, as Stengers (2005) says, aims to resist the probable. This is because, when we bring such actors, communities, schools of thought, practitioners, and so on together, even in the modest way we have here, we do not know what will emerge (see Michael, 2021). The bigger picture is about attending to such emergence as it unfolds. Second, insofar as our account is, perhaps frustratingly, vague, it avoids imposing our own particular take on how the process of the intersection among the various actors, communities, schools of thought, practitioners, and so on might be structured. The point is that each of these participants should be free to suggest their own process of interaction and intersection. Third, given that we too are embroiled in these enactments of the future, including those entailed in the unfolding of CE, we are in no position to provide a meta-narrative of the future (the future of CE futures). Instead, and fourth, the most we can offer is a resource—a set of sensibilities that loosen obligations and facilitate responsibilities in ways that bring these CE futures—both big and little—into “dialogue” (for want of a better term). Finally, our attachment to the probable does not mean we are unrealistic: We recognize that an ecology of practices and futures is a process; there are no straightforward outcomes and that it might lead to a proliferation of disjunctive CE futures as well as partial connection and possible consensus.

In staging this dialogue, we emphasize the interdisciplinary integration of CE practices that resonate deeply with the ethos of industrial ecology. By adopting the ecology of practices framework, we foster a holistic understanding of how varied disciplinary insights and practical engagements coalesce to shape sustainable futures. This approach not only seeks to bridge the gap between theory and practice but also highlights the critical role of operationalizing CE principles in real-world contexts, offering a pathway for diverse stakeholders to engage in the practical realization of sustainability goals.

Our paper thus contributes an adaptable and suggestive framework for analyzing and synthesizing the diverse and sometimes conflicting practices that define CE (but equally applied to other fields and discourses), advocating for a nuanced understanding that is both theoretically rich and grounded in practice. We hope that by articulating these contributions, we further enrich the discourse on CE, providing a foundational perspective that encourages continued exploration and collaboration.

## Data Availability

Data sharing is not applicable to this article as no datasets were generated or analyzed during the current study.

## Footnotes

1 1 We accept this is a partial and consistently evolving list. The purpose of the review is not to exhaust possibilities but highlight relevant examples of practice-led activities that could exemplify the ecology of practices approach.

2 2 Available online.
